# Supplementary material for: Elucidation of the Neuroprotective Effects of Astaxanthin Against Amyloid β Toxicity in the SH-SY5Y Human Neuroblastoma Cell Line
Source: Molecules. 2025 Nov 3;30(21):4271. doi: 10.3390/molecules30214271 (PMC12608137; doi:10.3390/molecules30214271)
Supplement: Supplementary file 1 [file molecules-30-04271-s001.zip › molecules-3936921-supplementary.pdf]

Supplementary Materials

Elucidation of the neuroprotective effects of astaxanthin against amyloid β toxicity in SH-SY5Y human neuroblastoma cell line

Sahithya Hulimane Ananda, Masahiro Kuragano* and Kiyotaka Tokuraku*

Division of Sustainable and Environmental Engineering, Muroran Institute of Technology, Mizumoto 27-1, Muroran, Hokkaido-050-8585, Japan

E-mail address: sahianand0104@gmail.com (S.H.A.)

* Corresponding author.
E-mail address: gano@muroran-it.ac.jp (M. K.)
E-mail address: tokuraku@muroran-it.ac.jp (K. T.)

Supplementary figures:

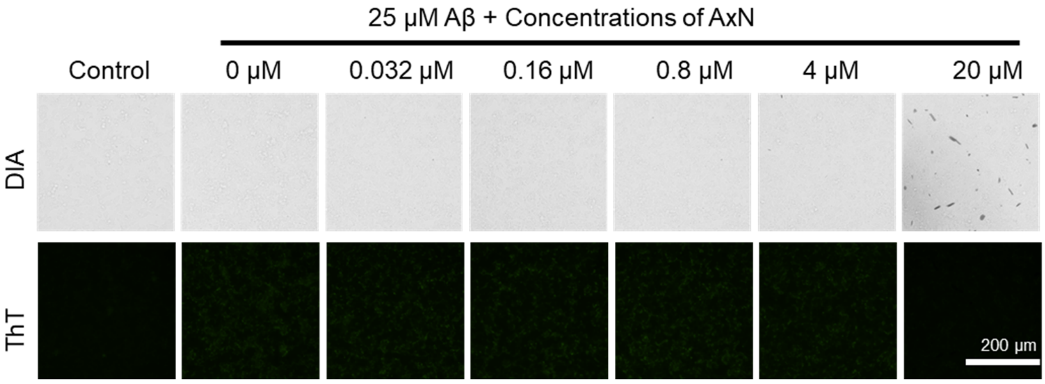

Supplementary figure S1. Effects of astaxanthin (AxN) on SH-SY5Y neuroblastoma cell motility. The raw images of Figure 5B. SH-SY5Y cells were co-incubated with 25 μM Aβ, 20 μM ThT and five concentrations of AxN and were observed by an inverted fluorescence microscope.

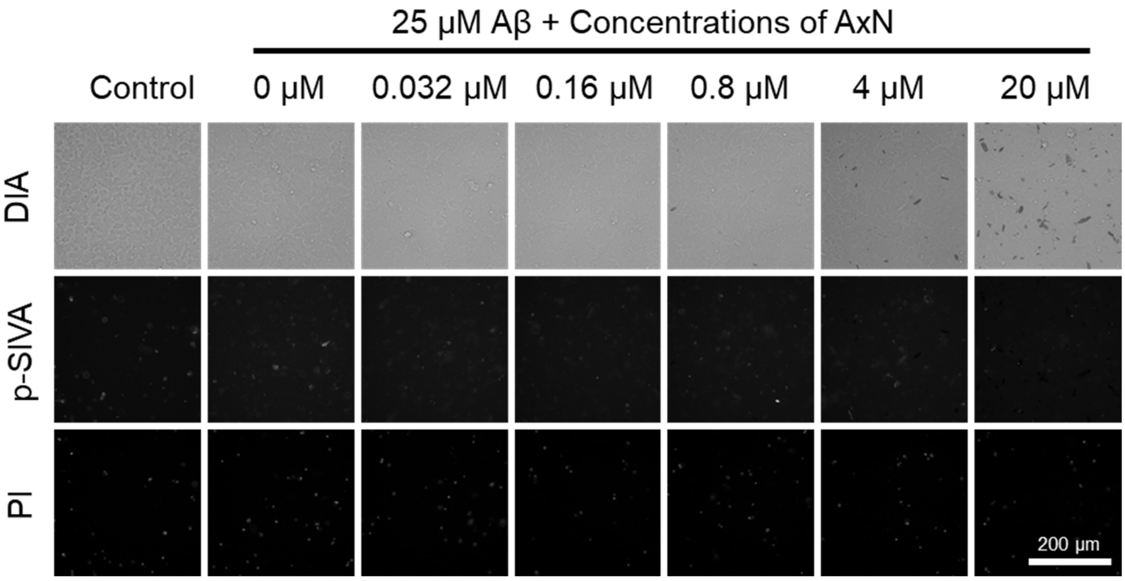

Supplementary figure S2. Effective prevention of early apoptosis but not late necrosis of SH-SY5Y neuroblastoma cells by astaxanthin (AxN). Raw images of Figure 4A. The morphological apoptosis and necrosis were determined by staining with pSIVA-IAMBD and propidium iodide (PI) respectively.

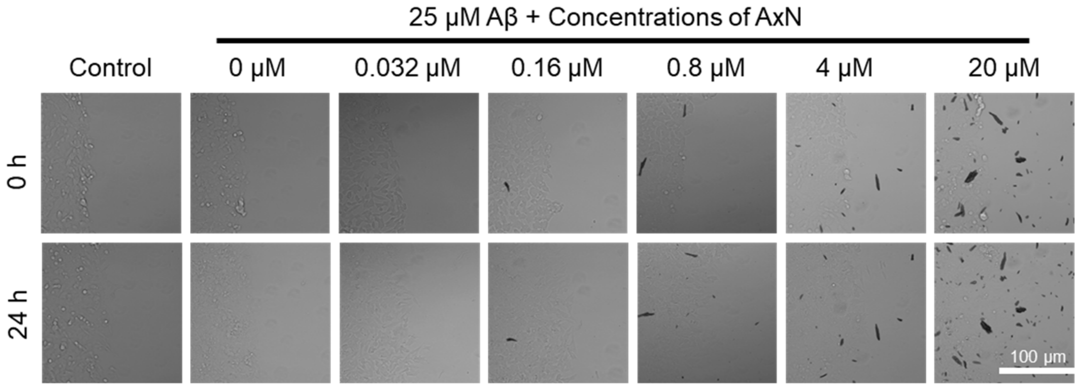

Supplementary figure S3 Effects of astaxanthin (AxN) on SH-SY5Y neuroblastoma cell motility. Raw images of Figure 5B. SH-SY5Y cells were co-incubated with 25 μM Aβ and five concentrations of AxN and were observed by an inverted fluorescence microscope.
